# Workplace genetic testing: which employees are likely to participate, what are their concerns with employer sponsorship, and which design features could reduce barriers and increase participation?

**DOI:** 10.3389/fgene.2024.1496900

**Published:** 2024-12-04

**Authors:** Forrest Briscoe, James H. Maxwell, Angel Bourgoin

**Affiliations:** ^1^ ILR School, Cornell University, Ithaca, NY, United States; ^2^ Environment and Health Group (United States), Cambridge, MA, United States; ^3^ JSI Research and Training Institute (United States), Atlanta, GA, United States

**Keywords:** ELSI, workplace, wellness, employee benefit, genetic testing (GT), population screening, workplace wellness program, genomic testing

## Abstract

Voluntary genetic testing (GT) leverages low-cost DNA sequencing and other testing methods to provide genetic risk screening for healthy individuals. Given the potential to prevent disease and promote health, some employers now offer GT as an employee benefit (workplace GT, or wGT), but participation remains low. To investigate facilitators and barriers to wGT participation, we conducted one of the first representative surveys of working U.S. adults on this topic (n = 958). We assessed factors that could influence participation, including: sponsoring entity (health provider or employer), program design, and individual demographics. Two-thirds (68%) of respondents indicated willingness to participate in some type of GT, but only half (49%) expressed willingness to participate through their employer. Women were 60% more willing to participate than men, and individuals with previous genetic testing experience were 143% more willing to participate than those without such experience. Across all demographic groups, certain GT program design features tended to increase or decrease willingness to participate. The ability to have one’s data deleted from the GT database increased willingness most often (true for 67% of respondents), while selling data to pharmaceutical companies decreased willingness most often (true for 63% of respondents).

## 1 Introduction

Some employers have recently begun offering voluntary genetic testing (GT) to their employees as a workplace benefit (McDonald et al., 2020; Sanghavi et al., 2021). By using genomic sequencing or other genetic testing technologies to screen healthy popluations for genetic risk factors related to cancer and other diseases, such workplace genetic testing (wGT) could promote health by informing people of their risks and recommending actions to mitigate them (Majumder et al., 2021; Khoury and Dotson, 2021; Charnysh, et al., 2024). While relatively few U.S. employers currently offer wGT (Business Group on Health, 2020; Cohn et al., 2023), this could change with advances in understanding of genetic disease risks, and as employers seek more ways to improve employee health, contain employee healthcare costs and maintain worker productivity (Kaiser Family Foundation, 2020). At the same time, while U.S. citizens are protected from discrimination based on their genetic information in health insurance and employment under the federal Genetic Information Nondiscrimination Act (GINA) and related state laws, many employees may still be concerned about privacy or forms of genetic discrimination that are not covered by current laws (Ajunwa, 2016; Joly et al., 2020). Hence it is important to identify factors that are likely to shape employees’ attitudes and decisions about whether to participate in such programs (Briscoe et al., 2023). These attitudes and behaviors are likely to change over time, as the role of genetic data in health care—and other domains of society—increases.

wGT programs are marketed in the United States by at least 12 different vendor firms, promoting their potential value to employers in improving workforce health and productivity, reducing employer and employee healthcare costs, and helping to attract, motivate, and retain employee talent (Deverka et al., 2020; author interviews). However, separate from the workplace, some employees may have access to similar testing through their health provider. At least seven health systems (such as Geisinger and Sanford Health) offer hGT directly to enrolled members, and two states (Nevada and Alabama) offer similar genetic screening programs to citizens in their states (Foss et al., 2022). Given these different types of GT sponsors, it is important to see if people are more or less likely to participate in employer-sponsored testing (wGT) compared with health provider testing (hGT).

From the employee’s point of view, wGT programs largely resemble direct-to-consumer (DTC) genetic testing services (e.g., 23andme). Individual employees who choose to participate receive a personalized report on their risk for diseases on the American College of Medical Genetics and Genomics (ACMG) list of clinically actionable diseases and conditions (Miller et al., 2022), as well as other conditions. Employees who are discovered to be at high genetic risk for cancers or other diseases may be directed to genetic counseling services. Reports may also steer employees toward enrollment in fitness, nutrition, or other wellness programs intended to help manage disease risk, or provided with educational materials. Some programs also include information about medication effectiveness to be discussed with their healthcare providers. Supporting the potential of wGT programs, Charnysh and colleagues (2024) recently reported that participants in one program who learned of their increased disease risk changed their subsequent health and utilization behaviors.

In recent interviews with employers offering or considering offering wGT to their employees, we uncovered potential barriers to employer adoption, including uncertainty about how much employees would participate in the programs. Such uncertainty accords with reported wGT participation rates of around 25% (Deverka et al., 2020; author interviews). This suggests that the practical viability of wGT programs—and their potential effectiveness in promoting health—may depend in part on attitudes in the broader U.S. workforce. How are employees likely to view and respond to wGT programs? Which groups of employees are more or less likely to participate? What design features might encourage or create barriers to participation?

To answer these questions, we conducted one of the first nationally representative surveys of working adults in the United States to assess factors that influence likeliness to participate in GT, including the sponsoring entity (healthcare provider vs employer) and individual demographic characteristics. In addition, drawing on earlier research and employee focus groups, we asked respondents how different wGT program design features would affect their likeliness to participate. We know of only one published survey on employee attitudes toward wGT, limited to employees of a biomedical research organization (Sanghavi et al., 2021).

## 2 Materials and methods

### 2.1 Study sample

Our survey was administered online by Qualtrics in collaboration with the National Opinion Research Center (NORC) during May 2023. Respondents were sourced from U.S. working adults included in NORC’s AmeriSpeak probability sample panel, which is designed to be representative of the broader U.S. population. In order to maintain representativeness, survey respondents were not screened for GT access or experience. The survey was completed by 1,016 working adults. Analysis for this article includes all respondents aged 18–64 (i.e., those who had not yet reached primary age for Medicare eligibility) for whom we had completed data on all survey items used (n = 958). The Pennsylvania State University Institutional Review Board approved this study after their review determined it to be exempt from ongoing IRB oversight (STUDY00013550).

### 2.2 Survey development

Our survey instrument was informed by prior published surveys of attitudes toward genetic testing in research and health provider contexts (Ewing, et al., 2015; Sanderson, et al., 2017). A common challenge for surveys involving new and technically sophisticated programs such as wGT is how to communicate basic information about the program and its benefits and risks such that respondents can provide informed responses. Following previous research, we conveyed this information within the survey instrument. With this information, we included comprehension check questions to increase the respondent’s incentive to examine the information. We tested and refined informational content to ensure clarity and neutral tone using Zoom focus groups with 13 students and pretests with 10 university employees.

Survey questions covering wGT attitudes were also informed by original focus group interviews with 21 employees of high-tech companies (which historically have been early adopters of employee benefits innovations). These focus groups explored employee views regarding wGT, and sought to identify specific design features that may affect participation. Themes that surfaced were related to privacy and data security, and how genetic data would be used. Participant concerns included: data sharing without consent; loss of privacy if a company were bought or if laws changed; financial risks for families if data was used by life insurance or other kinds of insurance companies; and use of data by law enforcement. In addition, even though participants were told about genetic anti-discrimination laws (e.g., GINA), some still worried about being dismissed if testing identified a health risk that is costly to treat, and about future employers accessing their results. Combining these findings with program design features reported by Briscoe et al. (2020), we developed survey items to assess the extent to which nine design features affect likeliness to participate.

### 2.3 Survey instrument

Respondents completed a 5–7 min survey on the Qualtrics platform. Before querying respondents about their attitudes toward GT, we collected baseline attitudes toward healthcare, employer, and government institutions, and then asked respondents to review information defining wGT and listing potential risks and benefits of participation. An example of information provided about wGT benefits is: “A look at select genes to better guide a screening and prevention plan for common hereditary cancers including breast, ovarian, and colorectal.” An example of information provided on wGT risks is: “Though there are some laws in place to protect against using genetic information as a basis for discrimination, there are gaps in protection for different types of insurance and employees in small businesses.” On the same page, respondents were also asked four basic yes/no comprehension check questions in order to focus their attention on the information provided.

Following this, respondents were asked to indicate their likeliness of participating in GT sponsored by (a) their employer, (b) their healthcare provider, or (c) a government agency, each on a scale from 1 (very unlikely) to 4 (very likely). Then, respondents indicated the extent to which nine design features affected their likeliness of participating in GT, on a scale from 1 (greatly decreases) to 5 (greatly increases). They were also asked about previous experience with direct-to-consumer (DTC) genetic testing, and likelihood of participating in DTC genetic testing in the future, on a scale from 1 (very unlikely) to 5 (very likely). Finally, respondents provided information about parental status and location of birth. For complete survey text, see Supplementary Material S1.

All respondents had also completed an earlier NORC questionnaire covering information about their personal, professional, and family background (see Supplementary Material S1). Survey responses and NORC AmeriSpeak questionnaire responses were matched and personal identifiers removed by Qualtrics/NORC prior to data analysis.

### 2.4 Data analysis

We conducted three broad sets of analyses. The first set of analyses focused on likeliness of GT participation in general (whether testing is sponsored by employer or health provider). The second set of analyses focused on employee skepticism, defined as respondents reporting they are unlikely to participate in wGT specifically. The third analysis focused on how different program design features affect likeliness to participate. Within the first two sets of analysis, we conducted univariate and multivariate logistic regressions to identify associations between respondents’ background characteristics and likeliness to participate. For these analyses, all background characteristics were dichotomized to facilitate analysis and interpretation. For the third set of analysis, we calculated the rates by which different design features would either increase or decrease likeliness to participate, and we used 2-sample t-tests to identify differences in those rates. All analyses and reported findings were conducted using STATA statistical analysis software (version 18.0) and incorporate representative population weights provide by NORC/Qualtrics.

## 3 Results

### 3.1 Respondent characteristics

The backgrounds of study respondents are broadly representative of the general U.S. adult working population. Nearly half were female (47.6%) and the majority identified as White (60.8%), followed by Hispanic (18.5%), Black (11.5%), and other (9.2%). Respondents’ ages ranged from 18 to 81 years (mean: 42.7 years). Most respondents had at least some college education (81.5%),worked as regular employees (89.5%), and were parents (63.4%). Approximately 12% identified as LGBTQ and 5% reported a disability. Approximately 11% of respondents were born outside the United States. Responses for political ideology followed a bell curve, with 45.7% of respondents identifying as moderate, and roughly even distributions on the liberal and conservative sides. Median household income for the sample was between $60,000 and $74,999.

### 3.2 Overall likelihood to participate

In this study, we use the terms workplace GT (wGT) and health provider GT (hGT) to differentiate between likeliness to participate in a hypothetical GT program sponsored by an individual’s employer *versus* their healthcare provider. Overall, two-thirds (68%) of respondents reported being likely to participate in GT (either wGT or hGT). Conversely, one-third (32%) of respondents reported being unlikely to participate in GT in either setting (Survey respondents were not asked about wGT experience, since pre-tests did not surface any reports of such experience.)

**FIGURE 1:**
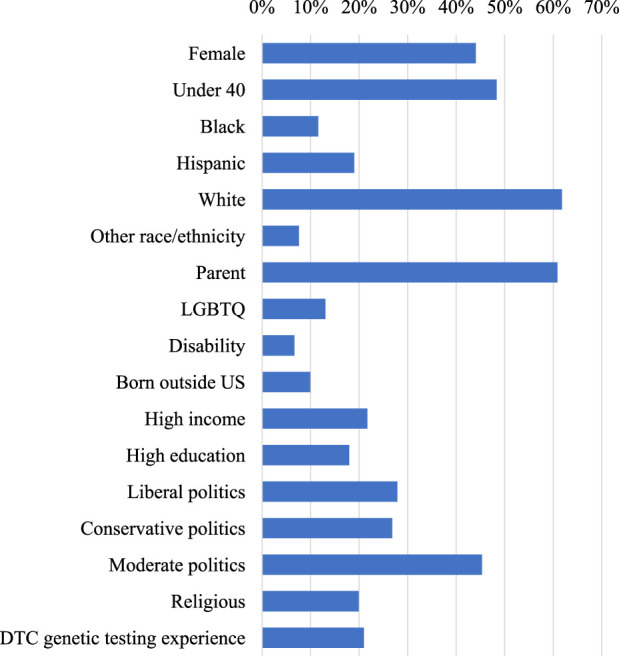
Percentage of respondents by background characteristic.

To investigate whether the likeliness of GT participation differed by respondent background, we evaluated the effect of self-reported gender, race, parental status, sexual orientation, disability status, country of birth, age, income, education, political beliefs, religiosity, and previous experience with DTC genetic testing. In Table 1, column 1 provides raw frequencies and weighted percentages of respondents with each background characteristic (also shown graphically in Figure 1), and columns 2 to 4 provide the results of univariate and multivariate analyses.

**TABLE 1:** Associations between respondent background characteristics and the likelihood of participating in GT (either wGT or hGT), among all respondents.

	1	2	3	4
Characteristic	N for group (weighted % of sample)	Univariate Results	Multivariate Results	Multivariate Results
Female	422	0.470*	0.481*	
	(47%)	(0.189)	(0.197)	
Under 40	463	0.274	0.332	
	(48%)	(0.188)	(0.193)	
Female over 40^a^	210	−0.052		0.146
	(24%)	(0.218)		(0.268)
Female under 40^a^	212	0.774**		0.906**
	(23%)	(0.238)		(0.276)
Male under 40^a^	251	−0.299		0.026
	(25%)	(0.209)		(0.255)
Black	111	−0.095	−0.392	−0.432
	(12%)	(0.277)	(0.284)	(0.283)
Hispanic	182	0.070	−0.148	−0.139
	(19%)	(0.250)	(0.286)	(0.285)
Other race	73	0.245	0.246	0.224
	(10%)	(0.365)	(0.413)	(0.413)
Parent	583	−0.023	0.069	0.079
	(62%)	(0.191)	(0.201)	(0.201)
LGBTQ	125	0.157	0.058	0.011
	(12%)	(0.254)	(0.284)	(0.289)
Disability	64	−0.099	−0.186	−0.188
	(5%)	(0.343)	(0.362)	(0.357)
Born outside US	95	0.219	0.286	0.290
	(12%)	(0.318)	(0.359)	(0.351)
High income	208	−0.086	−0.037	−0.019
	(23%)	(0.221)	(0.236)	(0.232)
High education	172	−0.236	−0.509*	−0.534*
	(18%)	(0.229)	(0.246)	(0.250)
Liberal	267	0.013	−0.360	−0.325
	(26%)	(0.205)	(0.238)	(0.240)
Conservative	257	−0.533**	−0.656**	−0.651**
	(28%)	(0.206)	(0.237)	(0.237)
Religious	191	−0.097	0.104	0.167
	(21%)	(0.226)	(0.250)	(0.246)
DTC genetic testing	201	0.889***	0.964***	0.982***
	(18%)	(0.242)	(0.258)	(0.260)
Constant			0.567*	0.689*
			(0.269)	(0.286)
N		958	958	958

Reported values in columns 2, 3 and 4 are coefficients with standard errors shown below in parentheses.

*p < 0.05; **p < 0.01; ***p < 0.001.

All results shown incorporate population weights.

^a^ For univariate regressions in column 2, coefficients reflect tests of these groups *versus* all other respondents. For multivariate regressions, coefficients reflect tests of these groups *versus* the base category of men ages 40 or older.

Four background characteristics have a statistically significant univariate association with the likeliness to participate in GT. First, the likeliness to participate is significantly lower among men. As an independent predictor, women are 60% more likely to express likeliness to participate in GT than men (Column 2, *p* < .05). After controlling for other factors using multivariate regression, this difference becomes slightly more pronounced, with women being 62% more likely to participate than men (Column 3, *p* < .05).

While age is not a significant predictor on its own, analysis reveals a striking pattern at the intersection of age and gender. As a group, women under age 40 are more than twice as likely (147% more likely) to participate in GT as men ages 40 or older (after controlling for other factors, Column 4, *p* < .01). Put differently, this implies that men ages 40 or older are less than half as likely (60% less likely) to participate as women under 40.

Likeliness to participate is also higher for individuals with DTC genetic testing experience. As an independent predictor, those with DTC genetic testing experience are more than twice as likely to participate in GT than those without such experience (143% more likely, Column 2, *p* < .001; 123% more likely after controlling for other factors, Column 3, *p* < .001).

Background characteristics that showed no significant univariate association with likelihood to participate include race (Black, Hispanic or Other race compared to the reference group of White), LGBTQ status, disability, being born outside the US, high income (over $125,000), religious attendance (attends services weekly), and education level (graduate or professional degree).

### 3.3 Preference for GT participation with health provider (hGT) over employer (wGT)

Respondents’ likelihood of participation in GT also varies based on the sponsoring entity. While nearly two-thirds of respondents (64%) reported being likely to participate in hGT, only half (49%) reported being likely to participate in wGT.

Most respondents who were likely to participate in wGT were also likely to participate in hGT, but the reverse was not as common. Specifically, of all those likely to participate in wGT, 91% were also likely to participate in hGT. But of those likely to participate in hGT, only 70% were also likely to participate in wGT. We therefore sought to further examine the subgroup of “employer skeptics” who were likely to participate in GT with their health provider but not their employer. This subgroup comprised 19% of all respondents.

**FIGURE 2:**
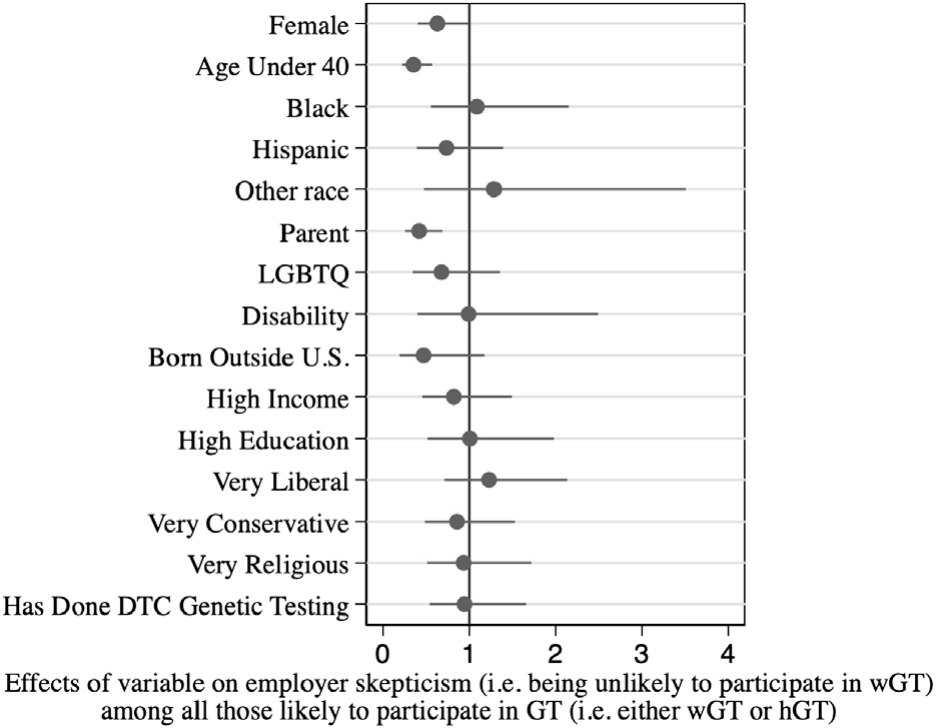
Associations between respondent background characteristics and employer skepticism*.

To better understand the employer skeptics, we also examined the background factors that predicted being in this subgroup, out of all those willing to participate in GT in general (either wGT or hGT). Results are shown in Table 2, and corresponding odds ratios are shown graphically in Figure 2.

**TABLE 2:** Associations between respondent background characteristics and employer skepticism (unlikely to participate in wGT), among those likely to participate in GT (either wGT or hGT).

	1	2	3
Characteristic	Univariate Results	Multivariate Results	Multivariate Results
Female	−0.435	−0.467*	
	(0.237)	(0.232)	
Under 40	−0.711**	−1.055***	
	(0.235)	(0.247)	
Female over 40^a^	0.289		−0.261
	(0.274)		(0.319)
Female under 40^a^	−0.928**		−1.571***
	(0.265)		(0.337)
Male under 40^a^	−0.072		−0.836*
	(0.272)		(0.329)
Black	−0.038	0.084	0.142
	(0.347)	(0.347)	(0.345)
Hispanic	−0.369	−0.311	−0.324
	(0.291)	(0.325)	(0.327)
Other race	0.163	0.250	0.259
	(0.498)	(0.512)	(0.515)
Parent	−0.463	−0.882***	−0.892***
	(0.243)	(0.257)	(0.259)
LGBTQ	−0.356	−0.394	−0.368
	(0.348)	(0.353)	(0.355)
Disability	0.145	−0.009	0.003
	(0.424)	(0.470)	(0.474)
Born outside US	−0.483	−0.764	−0.744
	(0.447)	(0.472)	(0.475)
High income	−0.033	−0.200	−0.184
	(0.308)	(0.305)	(0.304)
High education	0.024	0.007	0.017
	(0.305)	(0.344)	(0.346)
Liberal	0.047	0.207	0.178
	(0.255)	(0.281)	(0.281)
Conservative	−0.124	−0.155	−0.165
	(0.275)	(0.294)	(0.293)
Religious	−0.022	−0.069	−0.105
	(0.306)	(0.310)	(0.315)
DTC genetic testing	−0.061	−0.058	−0.078
	(0.264)	(0.286)	(0.283)
Constant		0.482	0.399
		(0.343)	(0.348)
N	634	634	634

Reported values in all models are coefficients with standard errors in parentheses.

*p < 0.05; **p < 0.01; ***p < 0.001.

All results shown incorporate population weights.

^a^ For univariate regressions, coefficients reflect tests of these groups *versus* all other respondents. For multivariate regressions, coefficients reflect tests of these groups *versus* the base category of men ages 40 or older.

The main findings from this analysis mirror those for overall GT participation: men and older respondents are more likely to be employer skeptics. As an independent predictor, women are 35% less likely to be employer skeptics than men (Column 1, *p* < .10; 37% less likely after controlling for other factors, Column 2, *p* < .05). Respondents under 40 are 55% less likely to be employer skeptics than respondents 40 or older (Column 1, *p* < .01; 65% less likely after controlling for other factors, Column 2, *p* < .001). Age and gender also combine to influence employer skepticism: women under age 40 are 79% less likely to be employer skeptics than men ages 40 and older (after controlling for other factors, Column 3, *p* < .001); put differently, men ages 40 and over are nearly five times as likely (381% more likely) to be employer skeptics, compared with women under age 40.

### 3.4 Design features that could reduce barriers to employee participation in wGT

As shown in Table 3, a majority of respondents indicate that the ability to later delete their data (67%), policies prohibiting data sale or sharing (61%), enhanced legal protections (60%), and control over how their data are used (55%) would increase their likelihood of participating in wGT. In addition, large numbers also indicated that restricting government/police access (48%) and implementing advanced cybersecurity systems (47%) would increase their likelihood of participating. In contrast, the wGT design features that most decrease likelihood of participation are: the ability to sell data to pharmaceutical companies (63%), depositing data in government databases (44%), and requiring links to health records (36%).

**TABLE 3:** Association of difference genetic testing (GT) program design features with respondent’s likeliness to participate.

Program design feature	% of respondents indicating feature increases likeliness to participate	% of respondents indicating feature does not change likeliness to participate	% of respondents indicating feature decreases likeliness to participate	Item text
Ability to delete data	66.6	29.4	4.0	Individuals have the right to request that their genetic testing data be deleted from the database at any time
No data sharing	60.9	32.8	6.3	Genetic testing data will not be sold, rented, or shared with any other organization
Legal protections	59.0	35.2	5.8	Genetic data will be treated in the same restrictive way as legally-protected medical records
Control of use	55.5	35.6	8.9	Individuals will be asked permission for each specific use of their genetic testing data in the future
Restricting government/police access	47.5	45.7	6.8	A warrant will be required for government and law enforcement to access genetic testing data
Cybersecurity	47.3	38.9	13.8	The best available security systems are used for all genetic testing and customer data
Linking to health records	21.1	42.4	36.5	The Genetic Wellness Program company requires access to your medical records, and these records will be linked to your genetic testing data
Depositing to a government database	15.1	42.1	42.8	Copies of genetic testing data (without individuals’ names) are deposited into a government database
Ability to sell data	13.5	23.7	62.8	Access to genetic testing data is sold to pharmaceutical firms (without requesting further permission from customers)

These preferences are highly consistent, regardless of background characteristics (see Supplementary Material S2). Across all groups, the design feature that most increases likelihood of participation is the ability to delete genetic data from databases, followed by policies prohibiting data sharing, legal protections, and control over how data are used. Likewise, the design feature that most decreases likelihood of participation is the ability to sell data to pharmaceutical companies across all groups except one. Notably, among Black employees, depositing genetic testing data in a government database is the wGT design feature that most decreases likelihood of participation (42%), followed closely by the ability to sell data to pharmaceutical companies (38%).

## 4 Discussion

Workplace genetic testing (wGT) is an innovative employee benefit currently offered by some employers, with aims of improving employee health and wellbeing, controlling employee-related healthcare costs, and improving workforce retention and productivity. In contrast to genetic testing in the health provider setting (hGT), wGT is made available to employees through independent employee benefit vendor companies. However, the ability of wGT to deliver desired results may depend in part on employee participation rates. Since individuals themselves are likely to vary in their attitudes toward wGT, this study of nationally representative employees explores factors that may influence the likelihood of participation.

We found that nearly 20% more respondents report being likely to participate in genetic testing (GT) with their health provider compared to their employer (68% hGT participation *versus* 49% wGT participation). This gap could reflect greater generalized public trust in health providers over employer organizations for handling sensitive medical information. Employers and wGT vendor companies may consider ways to close the gap, for example, by further integrating health providers such as genetic counselors into wGT programs that serve employees (Willard et al., 2024). Employers could also be well served by expanding access to hGT for their employees. For example, large employers could financially incentivize health plans and systems with which they contract to offer hGT services, whereby healthy patients are invited to participate by their doctors or a medical clinic, and actively encourage their workers to use those services (Foss et al., 2022).

### 4.1 Implications of wGT participation findings

Our wGT participation likelihood findings can be compared with the one previous published study on this topic, a survey by Sanghavi and colleagues (2021) of the employees of a biomedical research organization. In contrast to our findings, their results indicated more interest in workplace testing (70%) than health provider testing (54%). Higher wGT enthusiasm in their study context may reflect unusual employee-employer trust, or the unique nature of that biomedical research organization. Conversely, lower enthusiasm for wGT in our national sample may reflect greater generalized public trust in medical institutions (Hall et al., 2001) *versus* employers (Lucero and Allen, 1994). Our participation findings are also broadly consistent with initial results from Blasco et al. (2023) indicating 55% of national respondents were definitely or probably interested in wGT.

Importantly, we found participation likelihoods to vary across demographic groups. Like Sangavi and colleagues (2021), we found significantly lower participation likelihood among older workers. However, our study also found a lower likelihood among men compared with women, and especially low rates for older men. This gender gap is consistent with the skewed 75% female composition of community participants recruited recently to a state sponsored GT program (East et al., 2021). Applied to the employer context, this suggest wGT may have greater reach when deployed in companies and industries with younger and less male-dominated workforces.

Conversely, wGT may not be as effective in reaching populations of older male workers. For these groups, tailored educational and marketing materials could be considered to increase participation, for example, addressing the risks and benefits of genetic testing as perceived by older individuals (Waltz et al., 2018). While financial or other tangible incentives are used to increase employee participation in other types of corporate wellness programs (Kaiser Family Foundation, 2023), currently legal uncertainties and ethical concerns limit the use of such incentives for wGT (Chapman et al., 2020).

Our survey did not reveal statistically meaningful differences in likelihood of participation across racial or ethnic backgrounds. This contrasts with Briscoe and colleagues’ (2023) recent report from focus groups of elevated privacy and discrimination concerns among Black employees asked about wGT, and Abul-Husn et al. (2021) report of higher genetic screening interest among Hispanic/Latin individuals. More research is needed to understand potential barriers or enablers to wGT participation across racial and ethnic groups.

We also found that respondents with DTC genetic testing experience were much more likely to report interest in wGT. This indicates higher engagement among those already taking a proactive interest in personal health. This is consistent with traditional employee wellness programs, which also attract those already engaged in health promotion behavior (Beck et al., 2016; Hall et al., 2017). Of note, greater female participation is also common across traditional employee wellness programs (Beck et al., 2016; Hall et al., 2017).

### 4.2 Implications of wGT program design findings

We found certain wGT program design features to be associated with large increases in intent to participate, namely: the ability to delete one’s data, limits on data sharing, additional legal protections, and ongoing control over how one’s data are used. The design feature that most decreases willingness to participate across the board is selling data to pharmaceutical companies. It is also worth noting that unlike other employees, the design feature that decreases likelihood of participation most for Black employees is depositing genetic testing data in a government database. This difference could be related to a lack of trust in the government due to historic experiences of Black Americans, such as the Tuskegee Syphilis Study and the donation of Henrietta Lacks’ cancer cells (Corbie-Smith et al., 1999; Washington, 2006; Skloot, 2017).

These program design findings suggest several tensions that will merit attention as wGT programs are further developed by benefit vendor companies and implemented by employers. First, there is a tension between the re-use of genetic data and the extent of employee participation. The re-use of genetic data and health information from wGT programs can help advance biomedical research (Majumder et al., 2021; Mighton et al., 2022; NHGRI, 2023), and supports the commercial viability of wGT vendor companies. Yet our findings suggest that if wGT programs are designed to maximize data re-use, some employees will decline to participate–especially older men and those who have not yet undergone genetic testing. This wGT design tension parallels the current situation of DTC genetic testing companies and research biobanks, which often seek to re-use data without losing the trust of customers and publics (Laestadius, et al., 2017; Raz et al., 2020; Mladucky et al., 2021).

A second tension involves transparency. Recent commentaries call for wGT and DTC genetic testing to increase transparency in order to maintain stakeholder trust and increase participation (Hendricks-Sturrup and Lu, 2019; McDonald et al., 2020; Abitbol, et al., 2023). Logically, transparency is needed for employees and other stakeholders to know what is being done with their data. Yet our survey findings suggest that if employees learn that they lack control over their data, this awareness could actually reduce wGT participation.

Our findings regarding preferred wGT design features can be compared with preferences among DTC testing customers and research biobank participants (Sanderson et al., 2017; O’Doherty et al., 2021; Tiller et al., 2023). A common theme across these different settings is that the likelihood of participation goes up when people feel a greater ability to control their data and how it will be used. In our study, we found the largest barrier to participation is the sale of one’s genetic data to another company, consistent with previous studies in DTC testing and biobanking (Critchley et al., 2015; Briscoe et al., 2020; Mladucky et al., 2021), and reflective of general public concern with commercial use of genetic data (Tiller et al., 2023; Walshe et al., 2024).

Two of the wGT design features that we found increased the likelihood of participation the most–the right to request deletion of one’s data at a later time, and to approve or decline permission for future data re-uses–are consistent with a ‘dynamic informed consent’ model (Kaye et al., 2015). In that model, individuals have opportunities to approve or decline each subsequent sharing or re-use of their genetic data (Erlich, et al., 2014; Dankar et al., 2020). This contrasts with a ‘broad consent’ model in which a participant’s initial consent is designed to cover a wide range of later potential re-use scenarios for research or other purposes. While our findings suggest a wGT program designed for dynamic informed consent could expand participation, it would also add complexity and require vendor companies to maintain long-term contact with participants akin to the way employee retirement savings vendors function.

### 4.3 Study limitations

Like all research, this study has some limitations. First, we collected data in May 2023, when wGT programs were still relatively novel. As with any innovation, attitudes toward wGT may change over time as individuals become more familiar with them, and become more educated about genetic risks and genetic anti-discrimination legal protections (Willard et al., 2024). Second, our study used an online platform for survey administration, which may affect data quality. We sought to mitigate this limitation by using an academic research platform, a nationally representative probability sampling frame, and comprehension checks in our survey instrument. Third, some demographic information was not collected, including Asian, Indigenous, and Middle Eastern background, and religious affiliation.

### 4.4 Conclusion

Because workplace genetic testing (wGT) programs provide genetic testing in the context of an employee wellness benefit, they present unique opportunities and challenges. wGT programs have the potential to expand screening for actionable high-risk genetic diseases like cancer, and to address employer goals such as controlling workforce healthcare costs and improving health, employee retention and productivity. Yet our results suggest some employees do not trust their employer to sponsor this type of program, compared with having it sponsored by their health provider. Lower participation likelihood among specific employee subpopulations, and widespread concerns over data privacy and control, have important implications for the design of wGT programs to ensure more widespread dissemination and broad benefit.

## Data Availability

The raw data supporting the conclusions of this article will be made available by the authors, without undue reservation.
