# Supplementary material for: Workplace genetic testing: which employees are likely to participate, what are their concerns with employer sponsorship, and which design features could reduce barriers and increase participation?
Source: Front Genet. 2024 Dec 4;15:1496900. doi: 10.3389/fgene.2024.1496900 (PMC11652517; doi:10.3389/fgene.2024.1496900)
Supplement: Supplementary file 1 [file Table1.docx]

Supplementary Material

Genetic wellness programs: Which employees are likely to participate, what are their concerns with employer sponsorship, and which design features could reduce barriers and increase participation?

**Supplemental File S1: Survey Questionnaire Items**

***Screen 1. Baseline attitudes***

“How much confidence, if any, do you have in each of the following to act in your best interests?”

(A) - Your Employer(s)

(B) - Healthcare Providers

(C) - Government Agencies

Response options:

“A great deal of confidence”

“A fair amount of confidence”

“Not too much confidence”

“No confidence at all”

***Screen 2. Explanation of voluntary genetic testing benefits and comprehension checks***

Please review the information below:

A genetic wellness program is an employee benefit that provides personalized information about your hereditary risks that can potentially help you, your family members, and others lead a healthier life. Genetic testing will provide information on:

● Cancer risk: A look at select genes to better guide a screening and prevention plan for common hereditary cancers including breast, ovarian, and colorectal.

● Heart disease risk: A look at select genes associated with genetic forms of heart disease, including hereditary high cholesterol.

● Medication response: Analysis of genes associated with how the body may process certain medications.

● Fun insights: How well do you digest dairy products? What is your earwax type? Are you likely to love or hate cilantro?

● Family members’ hereditary risks: Your results could identify relatives who could benefit from genetic testing, and help future generations know what to look out for.

● Topics under medical research: Your data could be used in research studies to help identify hereditary conditions and improve medical treatment.

The following questions are used to check your comprehension of the material:

(A) “Can genetic testing help you understand your risk for heart disease?” (No or Yes)

(B) “Are genetic wellness programs employee benefits?” (No or Yes)

***Screen 3. Explanation of voluntary genetic testing risks and comprehension checks***

Employer-sponsored genetic testing has potential risks, such as the following:

● Inaccurate understanding: The results may be easy to misinterpret or could be based on a misapplication of the science.

● A burden of knowledge: A genetic test can leave you with information you’d prefer not to have about your family or about your risk for an incurable disease.

● Lower-quality counseling services: While companies that serve employer-sponsored genetic wellness programs often offer genetic counseling, a company counselor may be less familiar with your medical history than a counselor your doctor refers you to.

● Loss of control over your data: Once data about your genes is shared, it can be sold to others for uses you may not be aware of.

● Potential discrimination: Though there are some laws in place to protect against using genetic information as a basis for discrimination, there are gaps in protection for different types of insurance and employees in small businesses.

● Use by law enforcement: In some cases, your data could be used by law enforcement to identify suspects in crime scene investigations.

You can read more about these risks from the 2021 Consumer Reports article, “Read This Before You Buy a Genetic Testing Kit: At - home testing can offer an incomplete picture of disease risk, get ancestry wrong, and compromise privacy.”

The following questions are used to check to check your comprehension of the material:

(A) “Can genetic testing results be difficult to understand?” (No or Yes)

(B) “Can genetic testing data be used by law enforcement?” (No or Yes)

***Screen 4. Likeliness to participate***

“Based on what you have learned about genetic testing, how likely would you be to participate in a Genetic Wellness Program if it was sponsored by...”

(A) - Your employer?

(B) - Your healthcare provider?

(C) - a government agency?

Response options:

“Very likely”

“Likely”

“Unlikely”

“Very unlikely”

***Screen 5. Program design and likeliness to participate***

Organizations can have policies to govern the use, control, and safeguarding of genetic testing data. We are going to list some such policies.

Consider how knowing about this policy would affect your likeliness to participate in an employer-sponsored genetics wellness program:

(asked in randomized order)

A. The best available security systems are used for all genetic testing and customer data."

B. Access to genetic testing data is sold to pharmaceutical firms (without requesting further permission from customers)."

C. Copies of genetic testing data (without individuals' names) are deposited into a government database."

D. Individuals have the right to request that their genetic testing data be deleted from the database at any time."

E. The Genetic Wellness Program company requires access to your medical records, and these records will be linked to your genetic testing data."

F. Individuals will be asked permission for each specific use of their genetic testing data in the future."

G. Genetic testing data will not be sold, rented, or shared with any other organization."

H. A warrant will be required for government and law enforcement to access genetic testing data."

I. Genetic data will be treated in the same restrictive way as legally-protected medical records."

Response options:

“Greatly increases”

“Slightly increases”

“No change”

“Greatly decreases”

“Slightly decreases”

***Screen 6. Direct-to-consumer testing***

(A) “Have you ever participated in a direct-to-consumer genetics test before? Common vendors include 23andme, AncestryDNA, or FamilyTreeDNA.” (Yes or No)

(B) “How likely are you to take a direct-to-consumer genetics test?” (5 categories)

Response options:

“Very likely”

“Likely”

“No preference”

“Unlikely”

“Very unlikely”

***Screen 7. Background***

(A) “Are you a parent?” (Yes or No)

(B) “Were you born in the United States?” (Yes or No)

**NORC Panel Data Questionnaire items**

***Used in analysis***

1. Political ideology (5-level variable computed from multiple items)
2. “How often do you attend religious services?” (9 response categories)
3. “Does any disability, handicap, or chronic disease keep you from participating fully in work, school, household, or other activities?” (yes or no)
4. “Which of the following best represents how you think of yourself?” (Gay/Lesbian or gay; Straight, that is, not gay/Straight, that is, not lesbian or gay; Bisexual; Something else; I don’t know the answer)
5. Respondent gender (male, female, unknown)
6. Combined race/ethnicity (coded from multiple items: White non-Hispanic; Black, non-Hispanic; Other, non-Hispanic; Hispanic)
7. Education (5-level variable)
8. Household income (18-level variable)

***Not used in analysis (and had no effect on GT attitudes)***

1. Marital status (married, widowed, separated, divorced, never married)
2. Household members (and ages)
3. Region of the United States
4. Metropolitan or non-metropolitan region
5. Current employment status (2 response categories)
6. “Approximately how many paid employees does your entire company/organization have?” (11 response categories)
