# Supplementary material for: Workplace genetic testing: which employees are likely to participate, what are their concerns with employer sponsorship, and which design features could reduce barriers and increase participation?
Source: Front Genet. 2024 Dec 4;15:1496900. doi: 10.3389/fgene.2024.1496900 (PMC11652517; doi:10.3389/fgene.2024.1496900)
Supplement: Supplementary file 2 [file Table2.docx]

Supplementary Material

Genetic wellness programs: Which employees are likely to participate, what are their concerns with employer sponsorship, and which design features could reduce barriers and increase participation?

**Supplemental File S2: The Effects of wGT Program Design Features Are Consistent Across Demographic Groups**

**Supplemental Table 1. Percentage of each group indicating that a wGT program design feature would increase their likeliness to participate.**

| Characteristic | Program design feature | | | | | |
| --- | --- | --- | --- | --- | --- | --- |
|  | Ability to delete data | No data sharing | Legal protections | Control of use | Restricting gov’t/ police access | Cyber-security |
| Gender |  |  |  |  |  |  |
| Female | 68* | 65 | 62 | 60 | 49 | 48 |
| Male | 65* | 57 | 56 | 51 | 46 | 47 |
| Age |  |  |  |  |  |  |
| Older | 68* | 64 | 59 | 55 | 44 | 52 |
| Younger | 66* | 60 | 59 | 56 | 49 | 45 |
| Race |  |  |  |  |  |  |
| Black | 55* | 43 | 45 | 47 | 47 | 39 |
| Hispanic | 64* | 60 | 55 | 50 | 36 | 51 |
| White | 68* | 65 | 61 | 58 | 50 | 47 |
| Other | 74* | 63 | 70 | 57 | 54 | 51 |
| Parent |  |  |  |  |  |  |
| Yes | 66* | 59 | 57 | 54 | 46 | 46 |
| No | 68* | 64 | 62 | 58 | 50 | 50 |
| LGBTQ | 73* | 70 | 67 | 58 | 46 | 56 |
| Disability | 64* | 59 | 55 | 57 | 44 | 44 |
| Born outside US | 63* | 60 | 58 | 50 | 56 | 52 |
| Income |  |  |  |  |  |  |
| High | 69* | 68 | 65 | 60 | 50 | 50 |
| Low or middle | 66* | 59 | 57 | 54 | 47 | 47 |
| Education |  |  |  |  |  |  |
| Graduate | 77* | 68 | 65 | 62 | 55 | 53 |
| College or less | 64* | 59 | 58 | 54 | 46 | 46 |
| Political ideology |  |  |  |  |  |  |
| Very conservative | 59* | 56 | 50 | 46 | 39 | 35 |
| Liberal/moderate | 70* | 63 | 62 | 59 | 51 | 52 |
| Religiosity |  |  |  |  |  |  |
| Very religious | 65* | 53 | 54 | 52 | 42 | 48 |
| Not very religious | 67* | 63 | 60 | 56 | 49 | 47 |
| DTC experience | 72* | 71 | 70 | 68 | 56 | 53 |

* = Design feature that most increases likeliness to participate for each group.

**Supplemental Table 2. Percentage of each group indicating that a wGT program design feature would decrease their likeliness to participate.**

| Background characteristic | Program design characteristic | | |
| --- | --- | --- | --- |
|  | Linking to health records | Depositing in gov’t database | Selling data |
| Gender |  |  |  |
| Female | 35 | 41 | 63* |
| Male | 38 | 45 | 63* |
| Age |  |  |  |
| Older | 43 | 46 | 68* |
| Younger | 33 | 41 | 60* |
| Race |  |  |  |
| Black | 26 | 42* | 38 |
| Hispanic | 32 | 31 | 41* |
| White | 40 | 45 | 74* |
| Other | 35 | 55 | 64* |
| Parent |  |  |  |
| Yes | 34 | 43 | 63* |
| No | 40 | 43 | 63* |
| LGBTQ | 41 | 45 | 63* |
| Disability | 44 | 39 | 58* |
| Born outside US | 34 | 38 | 57* |
| Income |  |  |  |
| High | 43 | 52 | 78* |
| Low or middle | 35 | 40 | 58* |
| Education |  |  |  |
| Graduate | 43 | 48 | 79* |
| College or less | 35 | 42 | 59* |
| Political ideology |  |  |  |
| Very conservative | 35 | 44 | 61* |
| Liberal/moderate | 37 | 42 | 64* |
| Religiosity |  |  |  |
| Very religious | 30 | 39 | 60* |
| Not very religious | 38 | 44 | 64* |
| DTC experience | 42 | 40 | 64* |

*Design feature that most decreases likeliness to participate for each group.
